# Nomograms predicting survival and recurrence in colonic cancer in the era of complete mesocolic excision

**DOI:** 10.1002/bjs5.50167

**Published:** 2019-04-26

**Authors:** Y. Kanemitsu, D. Shida, S. Tsukamoto, H. Ueno, M. Ishiguro, S. Ishihara, K. Komori, K. Sugihara, Study Group for Nomogram of the Japanese Society for Cancer of the Colon and Rectum

**Affiliations:** ^1^ Department of Colorectal Surgery National Cancer Centre Hospital Saitama Japan; ^2^ Department of Translational Oncology and Graduate School, Tokyo Medical and Dental University Saitama Japan; ^3^ Department of Surgical Oncology Graduate School, Tokyo Medical and Dental University Saitama Japan; ^4^ Department of Surgical Oncology, School of Medicine The University of Tokyo Saitama Japan; ^5^ Department of Surgery National Defense Medical College Saitama Japan; ^6^ Department of Gastroenterological Surgery Aichi Cancer Centre Nagoya Japan

## Abstract

**Background:**

More extensive lymphadenectomy may improve survival after resection of colonic cancer. Nomograms were created predicting overall survival and recurrence for patients who undergo D2–D3 lymph node dissection, and their validity determined.

**Methods:**

This was a multicentre study of patients with colonic cancer who underwent resection with D2–D3 lymph node dissection in Japan. Inclusion criteria included R0 resection. A training cohort of patients operated on from 2007 to 2008 was analysed to construct prognostic models predicting survival and recurrence. Discrimination and calibration were performed using an external validation cohort from the Japanese colorectal cancer registry (procedures in 2005–2006).

**Results:**

The training cohort consisted of 2746 patients. Predictors of survival were: age (hazard ratio (HR) 1·04), female sex (HR 0·71), depth of tumour invasion (HR 1·15, 1·22, 2·96 and 3·14 for T2, T3, T4a and T4b respectively *versus* T1), lymphatic invasion (HR 1·11, 1·15 and 2·95 for ly1, ly2 and ly3 *versus* ly0), preoperative carcinoembryonic antigen (CEA) level (HR 1·21, 1·59 and 1·99 for 5·1–10·0, 10·1–20·0 and 20·1 and over *versus* 0–5·0 ng/ml), number of metastatic lymph nodes (HR 1·07), number of lymph nodes examined (HR 0·98) and extent of lymphadenectomy (HR 0·23, 0·13 and 0·11 for D1, D2 and D3 *versus* D0). Predictors of recurrence were: female sex (HR 0·82), macroscopic type (HR 3·82, 4·56, 6·66, 7·74 and 3·22 for types I, II, III, IV and V *versus* type 0), depth of invasion (HR 1·25, 2·66, 5·32 and 6·43 for T2, T3, T4a and T4b *versus* T1), venous invasion (HR 1·43, 3·05 and 4·79 for v1, v2 and v3 *versus* v0), preoperative CEA level (HR 1·39, 1·43, 1·56 and 1·85 for 5·1–10·0, 10·1–20·0, 20·1–40·0 and 40·1 or more *versus* 0–5 ng/ml), number of metastatic lymph nodes (HR 1·07) and number of lymph nodes examined (HR 0·98). The validation cohort comprised 4446 patients. The internal and external validated Harrell's C‐index values for the nomogram predicting survival were 0·75 and 0·74 respectively. Corresponding values for recurrence were 0·78 and 0·75.

**Conclusion:**

These nomograms could predict survival and recurrence after curative resection of colonic cancer.

## Introduction

Colonic cancer is common worldwide, and radical resection of the colon combined with regional lymph node dissection is the core of non‐metastatic colonic cancer treatment^1^. Expert series showing that more extensive lymphadenectomy is associated with excellent survival outcomes and low recurrence rates have stimulated interest in complete mesocolic excision (CME) with central vascular ligation (CVL) or extended lymph node dissection (D3)^2^, ^3^, ^4^, ^5^, ^6^. In Japan, colectomy with D3 lymph node dissection is performed routinely for T3 and T4 colonic cancer with low morbidity and mortality rates^4^, ^5^, ^6^. This dissection technique emphasizes anatomical lymph node dissection, and involves dissection of lymph nodes at the root of the tumour‐feeding artery and along the longitudinal length of the large intestine to be resected. In contrast, CME emphasizes identification of anatomical planes of surgical resection and CVL. Although these techniques differ in approach, the purpose and extent of lymph node dissection are similar^7^, except that the resected colon is shorter in the Japanese D3 procedure^3^.

Few nomograms predicting survival or recurrence of colonic cancer exist, and those that have been reported were based on a Western database^8^, ^9^. These nomograms have been validated for accuracy only by a data‐splitting method of the same Western internal database before the technique of CME with CVL and D3 lymph node dissection had emerged and where the extent of lymph node dissection was not specified^8^, ^9^.

The aim of the present study was to develop nomograms predicting survival and recurrence after curative colonic cancer resection based on D2–D3 lymph node dissection by combining clinicopathological variables using data from multiple institutions.

## Methods

This multicentre study was performed as part of a joint study by the Japanese Study Group for Outcome Prediction after Colorectal Cancer Surgery, whose members work at 19 major medical centres (4 cancer centres, 14 university hospitals and 1 teaching hospital) throughout Japan. Patients who underwent resection for stage I–III colonic cancer between 1 January 2007 and 31 December 2008 were eligible. Medical records were retrieved. Inclusion criteria were: primary colonic cancer, treatment with curative intent and R0 resection (no residual macroscopic or microscopic tumour). Exclusion criteria were: other malignancy, preoperative chemotherapy, distant metastases, missing data. These patients together formed the training cohort. To validate the data, an independent data set from the Japanese Society for Cancer of the Colon and Rectum (JSCCR) colorectal cancer registration was used^10^. This registry started in 1980 to present an overview of the actual state of surgical and pathological aspects of colorectal cancer treated in the leading hospitals in Japan. Results of patients who were treated at JSCCR‐member institutions, which comprise university hospitals, general hospitals and cancer centres, have been registered. This registry includes 6–7 per cent of all surgical cases of colorectal cancer in Japan^4^, ^11^. Patients in the validation cohort underwent colonic resection between 1 January 2005 and 31 December 2006, and satisfied the aforementioned inclusion criteria. The protocol was approved by the ethics committee of each hospital (institutional review board code 2013‐221).

### Data collection

Patient demographics, pathological characteristics, extent of lymphadenectomy, preoperative carcinoembryonic antigen (CEA) level, adjuvant chemotherapy and follow‐up data (duration of follow‐up, recurrence and survival) were collected. Tumour size was measured as the longest diameter. Macroscopic type was categorized as early colonic cancer with type 0 (superficial type), or colonic cancer with type I (polypoid type), II (ulcerated type with clear margin), III (ulcerated type with infiltration), IV (diffusely infiltrating type) or V (unclassified type) according to the criteria of the JSCCR *General Rules for Clinical and Pathological Studies on Cancer of the Colon, Rectum, and Anus* ^10^. The histological subtype was categorized as differentiated (well differentiated and moderately differentiated adenocarcinoma) or undifferentiated (poorly differentiated adenocarcinoma, signet ring cell carcinoma and mucinous adenocarcinoma). Depth of invasion was categorized as T1 (submucosa), T2 (muscularis propria), T3 (subserosa), T4a (serosa) or T4b (adjacent organ invasion). The degree of lymphovascular invasion was also classified according to the Japanese *General Rules* ^10^ as follows: no invasion (grade 0), minimal invasion (grade 1), moderate invasion (grade 2) and marked invasion (grade 3). The number of metastatic lymph nodes was categorized according to the node grouping of the eighth AJCC TNM classification (0, 1–2, 3–6, 7–15 or at least 16 nodes)^12^. According to the Japanese *General Rules* ^10^, nodes were divided into pericolic, intermediate and apical (D3) groups. The Japanese N category is based on both anatomical location and number of involved lymph nodes, classified as N0 (no evidence of lymph node metastasis), N1 (metastasis in 1–3 pericolic or intermediate lymph nodes), N2 (metastasis in 4 or more pericolic or intermediate lymph nodes) and N3 (metastasis in main or lateral lymph nodes). D2 dissection involves removal of pericolic and intermediate nodes, whereas D3 dissection involves removal of the main lymph nodes at the root of the regional artery in addition to D2 dissection. D2 or D3 dissection is recommended for patients with cT2 tumours, and D3 dissection for cT3 and cT4 lesions, or when lymph node metastasis is suspected^10^. Adjuvant chemotherapy was categorized as received or not received.

The discriminant value of the nomogram was compared with that of the AJCC TNM classification. In Japan, tumour deposits, which were introduced in the seventh edition, were not adopted in the national cancer staging manual edited by the JSCCR^10^. T categorization of tumour nodules in the mesocolic fat away from the leading edge of the tumour was done at the discretion of pathologists.

Follow‐up duration was measured from the date of surgery to the last follow‐up date, and information regarding survival status at last follow‐up was collected. At each hospital, postoperative follow‐up, according to the JSCCR guidelines^13^, consisted of serum tumour marker measurements every 3 months for the first 3 years, then every 6 months for 2 years; hepatic imaging (ultrasonography or CT) and chest X‐ray every 3–6 months; and colonoscopy every 2–3 years.

### Statistical analysis

#### *Construction of nomogram*

For nomogram construction, multivariable analysis was conducted using Cox proportional hazards (PH) regression. The PH assumption was verified by tests of correlations with time and examination of residual plots. To allow for non‐linear relationships, continuous variables were modelled with restricted cubic splines^14^ and were transformed to a form adequate for fitting the PH and linearity assumptions. The CEA level had a skewed distribution and was grouped into categories before modelling. Variables were selected by the forward stepwise selection method in the Cox PH regression model. Based on the predictive model with identified prognostic factors, a nomogram was constructed for predicting 3‐ and 5‐year overall survival (OS) or recurrence‐free survival (RFS). The nomogram assigned the probability of survival by adding up the scores identified on the points scale for each variable. The total score projected at the bottom indicated the probability of 3‐ and 5‐year survival.

#### *Validation of nomogram*

Nomogram validation consisted of analysis of discrimination and calibration using the validation set. Discrimination was evaluated using a concordance index (C‐index). This index provides the probability that, for two randomly selected patients, the patient with the worse outcome predicted by the nomogram indeed has an event before the other. Harrell's C‐index, which is appropriate for censored data, was used to evaluate discrimination^14^, ^15^. In general, a C‐index value greater than 0·75 is considered to represent relatively good discrimination. Calibration was performed by comparing the means of predicted survival with those of actual survival based on Kaplan–Meier estimates^16^ after grouping the nomogram‐predicted survival by decile.

Statistical analyses were performed using S‐plus® software version 8.0 (TIBCO Software, Palo Alto, California, USA). OS was calculated as the interval from primary surgery to death from any cause. RFS was defined as the time from surgery to any relapse or death from any cause or to the latest date at which relapse‐free status was confirmed. Censoring by the Kaplan–Meier method^16^ was performed for patients who did not experience the defined outcome. All *P* values were two‐sided. *P* < 0·050 was considered statistically significant.

## Results

The training cohort consisted of 2746 patients and the validation cohort included 4446 patients. Clinicopathological characteristics are shown in *Table* 1. Across the two cohorts, 34·4 and 61·3 per cent of patients underwent D2 and D3 lymph node dissection respectively.

**Table 1:** Demographic and clinicopathological variables in the training and validation cohorts

	Training cohort (*n* = 2746)	Validation cohort (*n* = 4446)
Age (years)*	68(11)	68(11)
Sex ratio (M : F)	1514 : 1232	2410 : 2036
Tumour location		
Caecum	298 (10·9)	
Ascending	604 (22·0)	
Transverse	407 (14·8)	
Descending	204 (7·4)	
Sigmoid	1233 (44·9)	
Tumour size (cm)*	3·5(2·4)	
Macroscopic type		
0	478 (17·4)	550 (12·4)
I	230 (8·4)	457 (10·3)
II	1933 (70·4)	3062 (68·9)
III	90 (3·3)	306 (6·9)
IV	3 (0·1)	8 (0·2)
V	12 (0·4)	63 (1·4)
Tumour differentiation		
Well or moderate	2594 (94·5)	
Poor or mucinous	150 (5·5)	
Other	2 (0·1)	
pT category		
pT1	526 (19·2)	654 (14·7)
pT2	394 (14·3)	653 (14·7)
pT3	1324 (48·2)	2271 (51·1)
pT4a	381 (13·9)	692 (15·6)
pT4b	121 (4·4)	176 (4·0)
Lymphatic invasion		
ly0	1254 (45·7)	1779 (40·0)
ly1	1116 (40·6)	1845 (41·5)
ly2	323 (11·8)	687 (15·5)
ly3	53 (1·9)	135 (3·0)
Venous invasion		
v0	1107 (40·3)	1849 (41·6)
v1	1120 (40·8)	1808 (40·7)
v2	408 (14·9)	644 (14·5)
v3	111 (4·0)	145 (3·3)
No. of LNs examined*	20·1(12·7)	18·8(12·9)
No. of metastatic LNs*	1·0(2·0)	1·0(2·1)
Preoperative CEA (ng/ml)		
0–5	1956 (71·2)	2998 (67·4)
5·1–10·0	402 (14·6)	752 (16·9)
10·1–20·0	202 (7·4)	351 (7·9)
20·1–40·0	99 (3·6)	166 (3·7)
≥ 40·1	87 (3·2)	179 (4·0)
TNM stage		
I	801 (29·2)	1093 (24·6)
IIA	825 (30·0)	1429 (32·1)
IIB	158 (5·8)	305 (6·9)
IIC	76 (2·8)	94 (2·1)
IIIA	113 (4·1)	189 (4·3)
IIIB	626 (22·8)	1062 (23·9)
IIIC	147 (5·4)	274 (6·2)
Extent of lymphadenectomy		
D0–1	134 (4·9)	185 (4·2)
D2	933 (34·0)	1528 (34·4)
D3	1679 (61·1)	2733 (61·5)
Adjuvant chemotherapy		
Yes	732 (26·7)	
No	2014 (73·3)	

Values in parentheses are percentages unless indicated otherwise;

* values are mean(s.d.). LN, lymph node; CEA, carcinoembryonic antigen.

Hazard ratios with 95 per cent confidence intervals for selected variables in Cox PH regression analyses are shown in *Tables* 2 and 3 respectively. In the multivariable model of OS, hazard ratios were significantly higher for older age, male sex, less extensive lymph node dissection, higher preoperative CEA level, greater depth of invasion, higher grade of lymphatic invasion, increased number of metastatic lymph nodes and decreased number of lymph nodes examined (*Table* 2).

**Table 2:** Selected variables according to the Cox proportional hazards regression model for overall survival

	Univariable analysis		Multivariable analysis	
	Hazard ratio	*P*	Hazard ratio	*P*
Age (years)*	1·05 (1·04, 1·06)	< 0·001	1·04 (1·03, 1·06)	< 0·001
Sex		0·018		0·004
M	1·00 (reference)		1·00 (reference)	
F	0·76 (0·61, 0·95)		0·71 (0·56, 0·89)	
Tumour location		0·651		
Caecum	1·00 (reference)			
Ascending	1·11 (0·75, 1·68)			
Transverse	1·03 (0·67, 1·60)			
Descending	1·16 (0·70, 1·91)			
Sigmoid	0·92 (0·64, 1·36)			
Tumour size (cm)*	1·01 (1·00, 1·01)	< 0·001	0·99 (0·99, 1·01)	0·923
Macroscopic type		< 0·001		0·445
0	1·00 (reference)		1·00 (reference)	
I	1·35 (0·66, 2·69)		1·11 (0·45, 2·66)	
II	3·44 (2·24, 5·59)		1·79 (0·84, 3·89)	
III	4·43 (2·27, 8·54)		1·64 (0·64, 4·19)	
IV	21·60 (5·09, 63·18)		3·25 (0·17, 18·80)	
V	5·92 (1·39, 17·28)		1·68 (0·34, 6·25)	
Tumour differentiation		< 0·001		0·617
Well	1·00 (reference)		1·00 (reference)	
Moderate	1·64 (1·29, 2·09)		1·24 (0·96, 1·61)	
Poor, signet or mucinous	2·19 (1·41, 3·29)		1·23 (0·74, 1·98)	
Extent of lymphadenectomy		0·003		< 0·001
D0	1·00 (reference)		1·00 (reference)	
D1	0·23 (0·06, 1·46)		0·23 (0·06, 1·46)	
D2	0·14 (0·04, 0·85)		0·13 (0·04, 0·78)	
D3	0·12 (0·03, 0·73)		0·11 (0·03, 0·98)	
Preoperative CEA (ng/ml)		0·004		0·009
0–5	1·00 (reference)		1·00 (reference)	
5·1–10·0	1·24 (0·90, 1·68)		1·21 (0·88, 1·64)	
10·1–20·0	1·53 (1·04, 2·18)		1·59 (1·08, 2·28)	
≥ 20·1	1·74 (1·22, 2·44)		1·99 (1·24, 3·09)	
pT category		< 0·001		< 0·001
T1	1·00 (reference)		1·00 (reference)	
T2	1·78 (1·01, 3·16)		1·15 (0·54, 2·52)	
T3	2·28 (1·40, 3·65)		1·22 (0·61, 2·63)	
T4a	5·85 (3·59, 9·87)		2·96 (1·30, 7·02)	
T4b	6·01 (3·37, 11·05)		3·14 (1·52, 6·89)	
Lymphatic invasion		< 0·001		0·003
ly0	1·00 (reference)		1·00 (reference)	
ly1	1·12 (0·86, 1·45)		1·11 (0·78, 1·33)	
ly2	1·16 (0·80, 1·65)		1·15 (0·67, 1·43)	
ly3	3·47 (1·88, 6·09)		2·95 (1·57, 5·28)	
Venous invasion		< 0·001		0·176
v0	1·00 (reference)		1·00 (reference)	
v1	1·61 (1·24, 2·11)		1·21 (0·90, 1·63)	
v2	2·47 (0·75, 2·58)		1·24 (0·59, 2·37)	
v3	15·91 (0·00, 65·65)		1·63 (0·96, 2·69)	
No. of LNs examined*	0·98 (0·97, 0·99)	0·031	0·98 (0·97, 0·99)	0·025
No. of metastatic LNs*	1·07 (1·03, 1·11)	< 0·001	1·07 (1·03, 1·11)	0·001
Adjuvant chemotherapy		0·506		
Yes	1·00 (reference)			
No	0·92 (0·73, 1·18)			

Values in parentheses are 95 per cent confidence intervals.

* Hazard ratios for factors analysed as a continuous variable are shown per unit increase. CEA, carcinoembryonic antigen; LN, lymph node.

**Table 3:** Selected variables according to the Cox proportional hazards regression model for recurrence‐free survival

	Univariable analysis		Multivariable analysis	
	Hazard ratio	*P*	Hazard ratio	*P*
Age (years)*	0·99 (0·98, 1·01)	0·708		
Sex		0·025		0·045
M	1·00 (reference)		1·00 (reference)	
F	0·86 (0·71, 0·99)		0·82 (0·66, 0·99)	
Tumour location		0·431		
Caecum	1·00 (reference)			
Ascending	0·77 (0·55, 1·09)			
Transverse	0·79 (0·55, 1·16)			
Descending	0·99 (0·65, 1·51)			
Sigmoid	0·79 (0·58, 1·08)			
Tumour size (cm)*	1·01 (1·00, 1·02)	< 0·001	0·98 (0·97, 1·11)	0·175
Macroscopic type		< 0·001		0·046
0	1·00 (reference)		1·00 (reference)	
I	6·81 (2·88, 18·70)		3·82 (1·23, 13·43)	
II	15·46 (7·56, 39·11)		4·56 (1·58, 15·47)	
III	33·19 (14·87, 88·25)		6·66 (2·14, 23·82)	
IV	47·08 (6·89, 204·41)		7·74 (0·38, 56·51)	
V	14·66 (2·15, 63·63)		3·22 (0·41, 17·74)	
Tumour differentiation		< 0·001		0·228
Well	1·00 (reference)		1·00 (reference)	
Moderate	1·95 (1·57, 2·43)		1·29 (0·92, 1·63)	
Poor, signet or mucinous	2·27 (1·50, 3·32)		1·01 (0·64, 1·54)	
Extent of lymphadenectomy		< 0·001		0·449
D0	1·00 (reference)		1·00 (reference)	
D1	0·78 (0·46, 1·44)		0·23 (0·18, 1·28)	
D2	0·55 (0·29, 0·88)		0·57 (0·31, 1·14)	
D3	0·23 (0·05, 0·73)		0·65 (0·36, 1·28)	
Preoperative CEA (ng/ml)		< 0·001		0·003
0–5	1·00 (reference)		1·00 (reference)	
5·1–10·0	2·07 (1·60, 2·67)		1·39 (0·99, 1·91)	
10·1–20·0	2·54 (1·82, 3·46)		1·43 (1·10, 1·85)	
20·1–40·0	2·86 (1·84, 4·24)		1·56 (0·99, 2·34)	
≥ 40·1	4·19 (2·82, 6·02)		1·85 (1·22, 2·71)	
pT category		< 0·001		< 0·001
pT1	1·00 (reference)		1·00 (reference)	
pT2	2·36 (1·04, 5·61)		1·25 (0·88, 5·52)	
pT3	9·89 (5·39, 20·84)		2·66 (1·16, 7·24)	
pT4a	27·19 (14·69, 57·57)		5·32 (2·14, 15·30)	
pT4b	23·95 (12·12, 52·81)		6·43 (2·76, 17·69)	
Lymphatic invasion		< 0·001		0·132
ly0	1·00 (reference)		1·00 (reference)	
ly1	1·62 (1·29, 2·05)		1·01 (0·79, 1·30)	
ly2	2·57 (1·93, 3·41)		1·04 (0·75, 1·43)	
ly3	7·01 (4·49, 10·52)		1·88 (0·97, 3·16)	
Venous invasion		< 0·001		< 0·001
v0	1·00 (reference)		1·00 (reference)	
v1	2·53 (1·94, 3·33)		1·43 (1·09, 1·91)	
v2	2·85 (1·43, 5·14)		3·05 (2·50, 6·87)	
v3	7·03 (0·00, 38·90)		4·79 (0·00, 29·92)	
No. of LNs examined*	0·97 (0·96, 0·99)	0·045	0·98 (0·97, 0·99)	0·006
No. of metastatic LNs*	1·17 (1·15, 1·20)	< 0·001	1·07 (1·03, 1·11)	< 0·001
Adjuvant chemotherapy		0·624		
Yes	1·00 (reference)			
No	0·97 (0·65, 1·87)			

Values in parentheses are 95 per cent confidence intervals.

* Hazard ratios for factors analysed as a continuous variable are shown per unit increase. CEA, carcinoembryonic antigen; LN, lymph node.

For RFS, hazard ratios in the multivariable model were significantly higher for male sex, advanced macroscopic type, higher preoperative CEA level, greater depth of invasion, higher grade of venous invasion, increased number of metastatic lymph nodes and decreased number of lymph nodes examined (*Table* 3).

**Figure 1:**
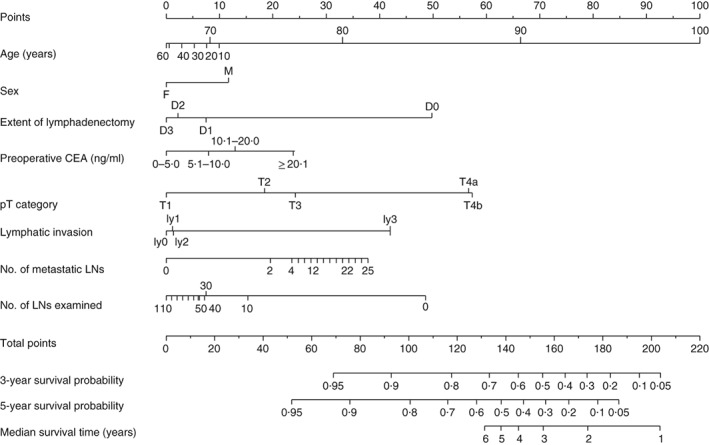
Prognostic nomogram for predicting overall survival of patients with colonic cancer. The nomogram can assign the probability of survival by adding up the scores identified on the points scale for each variable. The total score projected to the bottom scale indicates the probability of 3‐ and 5‐year survival. CEA, carcinoembryonic antigen; LN, lymph node

**Figure 2:**
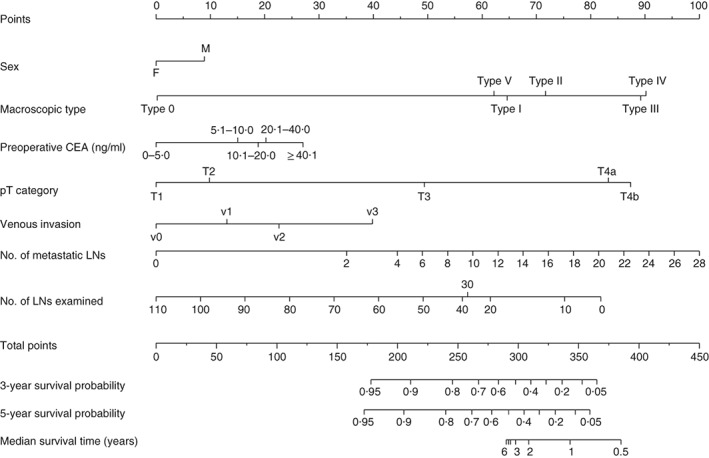
Prognostic nomogram for predicting recurrence‐free survival of patients with colonic cancer. The nomogram can assign the probability of survival by adding up the scores identified on the points scale for each variable. The total score projected to the bottom scale indicates the probability of 3‐ and 5‐year survival. CEA, carcinoembryonic antigen; LN, lymph node

**Figure 3:**
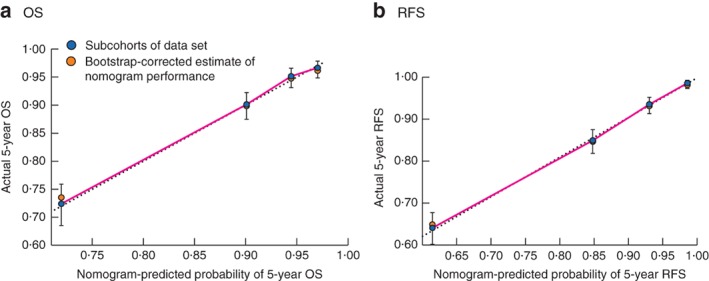
Calibration of the nomogram in the training cohort. **a** Five‐year overall survival (OS) and **b** 5‐year recurrence‐free survival (RFS). Actual survival rates with 95 per cent confidence intervals were calculated by Kaplan–Meier analysis. The dotted line represents the ideal reference line where predicted survival corresponds to actual survival

Median follow‐up was 61·1 (i.q.r. 35·5–69·4) months for recurrence and 61·6 (48·9–70·6) months for survival in the training set, and 64·2 (31·3–83·8) and 68·5 (44·2–84·7) respectively in the validation set. Five‐year OS rates were 88·7 and 85·6 per cent in the training and validation sets respectively, with corresponding RFS rates of 85·1 and 84·9 per cent. To evaluate the OS and RFS of patients with stage I–III colonic cancer, nomograms were constructed based on independent variables for OS (*Fig*. 1) and RFS (*Fig*. 2) in the multivariable Cox regression model. Harrell's C‐index values for the OS and RFS nomograms were 0·747 (95 per cent c.i. 0·697 to 0·788) and 0·781 (0·732 to 0·821) respectively. The calibration curves for the two nomograms are shown in *Fig*. 3. Actual survival corresponded closely with predicted survival and was always within the 10 per cent margin of error. These curves reveal the concordance in the original cohort between the nomogram forecast and actual observations for 5‐year OS and RFS.

### Validation

**Figure 4:**
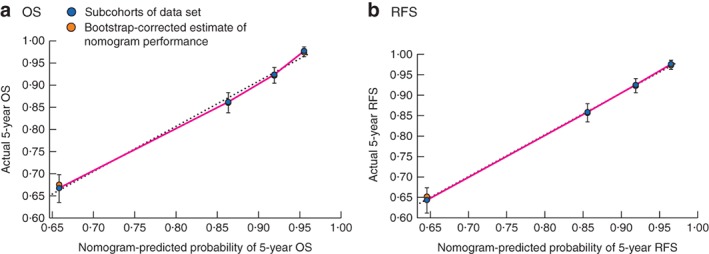
Calibration of the nomogram in the validation cohort. **a** Five‐year overall survival (OS) and **b** 5‐year recurrence‐free survival (RFS). Actual survival rates with 95 per cent confidence intervals were calculated by Kaplan–Meier analysis. The dotted line represents the ideal reference line where predicted survival corresponds to actual survival

**Figure 5:**
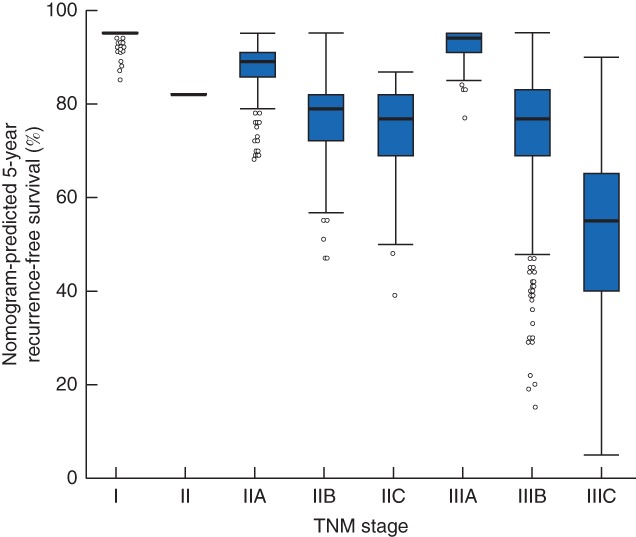
Predicted stage‐specific recurrence‐free survival based on the eighth AJCC classification. Median value (bold line), box (i.q.r.), and range (error bars) excluding outliers (circles) are shown

In the validation set, Harrell's C‐index values for the OS and RFS nomogram were 0·738 (95 per cent c.i. 0·699 to 0·777) and 0·752 (0·708 to 0·795) respectively. The nomogram also predicted OS and RFS better than chance for the external data set. Calibration plots suggested that the nomogram was well calibrated for all predictions (*Fig*. 4). Discrimination of the nomograms was compared with that of the eighth AJCC TNM classification. Each nomogram was superior to that of the eighth AJCC TNM classification, which had C‐index values of 0·631 (0·591 to 0·673) for OS and 0·554 (0·521 to 0·597) for RFS. *Fig*. 5 illustrates the 5‐year RFS predicted by the nomogram for each stage of the eighth AJCC TNM classification. Variation in predicted survival could be identified in each TNM stage. Predicted survival was more variable for higher stages.

## Discussion

The nomograms in this study provide significantly better discrimination than the eighth AJCC TNM classification, and allow an individualized prediction of survival and recurrence that may be used to inform treatment planning and patient care.

Until now, nomograms predicting the prognosis of patients with stage I–III colonic cancer have had major limitations, because they were constructed from data collected before the technique of CME with CVL had emerged and the extent of lymph node dissection was not specified^8^, ^9^. In contrast to these two studies^8^, ^9^, extent of lymphadenectomy, preoperative CEA level and lymphatic invasion were included in the present OS nomogram, and macroscopic type, venous invasion, number of metastatic lymph nodes and number of lymph nodes examined in the RFS nomogram. Although previous studies^17^, ^18^ have also shown that a raised serum CEA level before treatment is associated with poor prognosis in patients with colorectal cancer, the optimum cut‐off value of CEA has not been defined. Ideally, the predictor should be a continuous variable to maximize the amount of information that it can convey^19^. Although continuous variables can preserve information more than categorical variables, drawing lines to points in the nomogram and summing points can be ambiguous and cumbersome. In this study, preoperative CEA was categorized by using statistical methods to fit the PH and linearity assumptions. The number of lymph nodes examined, which was included in both of the present nomograms, has been shown to correlate with outcomes in other studies^11^, ^20^, ^21^, ^22^. The mean numbers of lymph nodes examined in this study were 20·1 and 18·8 in the training and validation sets respectively. These numbers were higher than that in Weiser and colleagues' study^9^, where the number of examined lymph nodes was 12·9. Regarding macroscopic type of cancer, some studies^23^, ^24^ have shown that macroscopic type may reflect tumour behaviour. Types III (ulcerated type with infiltration) and IV (diffusely infiltrating type) are invasive phenotypes that carry a worse prognosis in terms of RFS than other macroscopic types.

The extent of lymphadenectomy was established as one of the important prognostic factors in the OS nomogram. Recently, the extent of lymph node dissection was reported to have a positive impact on survival of patients with curatively resected colorectal cancer without distant metastasis^2^, ^3^, ^4^, ^25^. CME with CVL and Japanese D3 dissection proved superior to previously reported techniques^3^. A multicentre cohort study^25^ in Denmark revealed that CME with CVL may improve long‐term oncological outcomes by 6–14 per cent compared with standard European surgery for each of the AJCC pathological stage I–III colonic cancers^25^. The Japan Clinical Oncology Group 0404 trial^6^ also had the advantage that it was an RCT that aimed to evaluate whether laparoscopic D3 dissection was non‐inferior to open D3 dissection. OS in both groups was similar, and better than the expected 5‐year OS rate of 90 per cent.

External validation of the present results is essential. The high C‐index values in this study indicate a high level of predictive accuracy. There are, nevertheless, limitations. Patient co‐morbidity was not included in these nomograms. It is expected that co‐morbidity would affect OS. The time span for the data set was more than 10 years. This raised the question of whether these nomograms can be applied to current patients. In most institutions in Japan, however, indications for surgery, systemic treatment, surgical strategy for D2–D3 lymph node dissection and pathological examination have not changed in the past decade. Novel pathological and molecular markers, such as perineural infiltration, mismatch repair status and *RAS*/*RAF* mutational status, were not available at the time of this study. Future studies could see if these variables might be included in nomograms to predict survival and recurrence after curative resection of colonic cancer with advanced surgical techniques for lymphadenectomy.
